# Adult brain cancer incidence patterns: A comparative study between Japan and Japanese Americans

**DOI:** 10.1002/ijc.35374

**Published:** 2025-02-21

**Authors:** Byron Sigel, Diana R. Withrow, Lene H. S. Veiga, Eiko Saito, Tomohiro Matsuda, Kota Katanoda

**Affiliations:** ^1^ Department of Medicine Washington University School of Medicine St. Louis Missouri USA; ^2^ Department of Primary Care Health Sciences University of Oxford Oxford UK; ^3^ Radiation Epidemiology Branch, Division of Cancer Epidemiology and Genetics National Cancer Institute Rockville Maryland USA; ^4^ Sustainable Society Design Center, Graduate School of Frontier Sciences The University of Tokyo Chiba Japan; ^5^ National Cancer Center Institute for Cancer Control Tokyo Japan

**Keywords:** brain and central nervous system tumors, incidence rates, Japanese ancestry, migrant study, non‐Hispanic Whites

## Abstract

Adult primary brain and central nervous system (CNS) cancers, though comprising only about 4% of new cancer diagnoses, significantly impact morbidity and mortality due to their low survival rates. Globally, brain and CNS tumor incidence varies considerably, with the United States exhibiting one of the highest rates and Japan among the lowest worldwide. In the United States, incidence rates differ by race, with higher rates in non‐Hispanic whites (NHW) and lower rates in Asian Americans and Pacific Islanders (AAPI). This study examines the incidence of malignant CNS tumors in Japan and Japanese Americans, comparing these groups to NHW and AAPI populations in the United States. We estimated age‐standardized incidence rates (ASR) of brain and CNS tumors among adults using data from the Monitoring of Cancer Incidence in Japan (MCIJ) and the U.S. Surveillance, Epidemiology, and End Results (SEER)‐9 registries from 2007 to 2014. Incidence rates were stratified by age, sex, and specific CNS tumor subtypes. Incidence rates of CNS tumors among Japanese (ASR: 3.66, 95% CI: 3.56–3.76) and Japanese Americans (ASR: 2.5, 95% CI: 2.13–3.05) were lower than among NHW (9.43, 95% CI, 9.31–9.56) and AAPI populations (ASR: 4.13, 95% CI: 3.94–4.33) in the United States. The same pattern was observed for CNS tumor subtypes and across age groups and sex. This study supports a genetic component in the risk of brain and CNS tumors, a cancer type with largely unknown etiology. By comparing incidence rates across populations, it contributes to understanding the balance of genetic and environmental risk factors in the development of these cancers.

## Graphical abstract

What's new?

The risk factors for brain tumors remain largely unknown. Building on the legacy of migration studies, this study compared the incidence of adult malignant brain and central nervous system tumors in Japan and among Japanese Americans and other ethnic populations in the United States. The results revealed lower incidence rates in both the Japanese and the Japanese American populations compared with the non‐Hispanic White population in the United States. This pattern remained consistent across sex and age groups. The findings suggest that genetic factors play a stronger role than environmental factors in the brain and central nervous system tumor etiology.

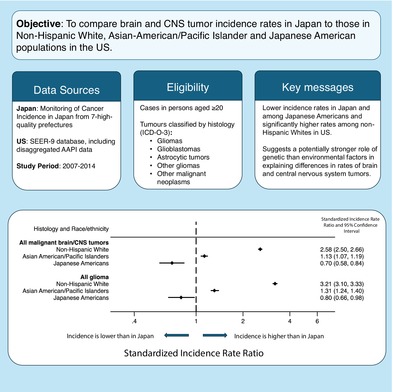

## 1 INTRODUCTION

Adult primary brain and central nervous system (CNS) cancers are relatively rare, accounting for about 4% of new cancer diagnoses. Nevertheless, they cause significant morbidity and mortality, with many subtypes having low survival rates. While there have been great efforts to understand the etiology of brain and CNS tumors, risk factors remain largely unknown. The only established risk factors are genetic predisposition and exposure to moderate to high levels of ionizing radiation.^1^ Other environmental and lifestyle risk factors may play a role, and the relative contributions of genetic and environmental factors to CNS risk are unknown.

Globally, there is significant variation in the incidence rate of brain and CNS tumors, with higher incidence rates in high‐income countries.^2^ In the United States, incidence rates of CNS tumors overall have been estimated to be 6.2 and 4.7 per 100,000 person‐years among men and women, respectively, whereas in Japan they have been estimated to be 3.1 and 2.4, respectively.^2^ Within the United States, incidence rates are heterogeneous by race, with higher rates in NHW and lower rates in Asian Americans and Pacific Islanders (AAPI),^3^ which include the Japanese ethnic population in the United States.

Migrant studies, which compare disease rates in immigrants to those in the countries of origin and settlement, have long been used to provide insight into the balance of genetic versus environmental causes of disease and to generate hypotheses about what environmental or lifestyle factors may increase risk.^4^

Given the limited understanding of brain tumor etiology and the significant global variation in risk, our study aims to build on the legacy of migration studies by evaluating the incidence of adult malignant brain and CNS tumors in Japan and among the Japanese ethnic population in the United States. We also compare these findings to the incidence rates among NHW and AAPI populations in the United States.^5^

We used data from the MCIJ database and a specialized SEER database, which provide cancer data stratified by specific subpopulations within the AAPI category.

## 2 METHODS

### 2.1 Data sources

U.S. cancer incidence data, which included disaggregated rates for 11 race/ethnicity categories within the AAPI group (including Japanese) was obtained from a specialized SEER database created by the U.S. National Cancer Institute.^6^, ^7^ We used data from the geographic areas covered by the 9 original SEER registries located in: Atlanta, Connecticut, Detroit, Hawaii, Iowa, New Mexico, San Francisco‐Oakland, Seattle‐Puget Sound, and Utah. SEER‐9 data was utilized because this set of registries is the only one for which the specialized dataset with narrower AAPI populations is available. More specific AAPI populations were determined based on self‐reported ancestry, with population denominators estimated by extrapolating data from the 1990 and 2000 censuses.^7^ We restricted the analysis to the years 2007 to 2014 since high‐quality comparison data had only been available in Japan since 2007.

Cancer incidence rates in Japan between 2007 and 2014 were obtained from seven prefecture‐level population‐based registries participating in the MCIJ project within the National Cancer Center Japan.^8^, ^9^ In 2006, Japan enacted the Cancer Control Act and in 2007 the Basic Plan to Promote Cancer Control Programs. Together, these programs led to an improvement in data quality. Seven prefectures were selected based on the following quality metrics: <20% of cases registered as death certificate only, fewer than 30% of cases identified through death certificate notification, and over 50% of cases microscopically verified. These metrics are not as strict as other guidelines (such as Cancer Incidence in Five Continents) but were selected to balance having a representative sample of national cases and ensuring these were of sufficiently high quality. While these thresholds were used at the prefecture level, in the combined data, the percentage of death certificates only does not exceed 10% and microscopically verified cases constitute more than 70% of the total cases (Table 1), making it closer to international standards.^10^

**TABLE 1:** Characteristics of malignant cases of brain and CNS tumors, 2007–2014.

Characteristic	U.S. SEER 9			
	Non‐Hispanic White		Asian American/Pacific Islanders	
	*N*	%	*N*	%
Total	24,405	100	1805	100
Sex				
Male	13,907	57.0	983	54.5
Female	10,498	43.0	822	45.5
Calendar year of diagnosis				
2007–2008	5999	24.6	414	22.9
2009–2010	6057	24.8	455	25.2
2011–2012	6183	25.3	441	24.4
2013–2014	6166	25.3	495	27.4
Age at diagnosis				
20–34 years	2099	8.6	235	13.0
35–49 years	3566	14.6	391	21.7
50–64 years	7999	32.8	495	27.4
65–84 years	9138	37.4	583	32.3
85+ years	1603	6.6	101	5.6
Stage				
Localized	18,238	74.7	1250	69.3
Regional	3847	15.8	330	18.3
Distant	364	1.5	43	2.4
Unknown	1956	8.0	182	10.1
Mode of diagnosis				
Cases microscopically verified	21,094	86.4	1531	84.8
Histology	21,068	86.3	1529	84.7
Cytology	20	0.1	2	0.1
Clinical examination or diagnosis (including radiography)	2481	10.2	199	11.0
Unknown or other modes of diagnosis	830	3.4	75	4.2
Source of information				
Notification	24,007	98.4	1778	98.5
Death certificate only	398	1.6	27	1.5

Characteristic	U.S. SEER 9		Japan	
	Japanese Americans		Overall	
	*N*	%	*N*	%
Total	141	100	5832	100
Sex				
Male	70	49.6	3173	54.4
Female	71	50.4	2659	45.6
Calendar year of diagnosis				
2007–2008	35	24.8	1325	22.7
2009–2010	41	29.1	1413	24.2
2011–2012	25	17.7	1505	25.8
2013–2014	40	28.4	1589	27.2
Age at diagnosis				
20–34 years	14	9.9	489	8.4
35–49 years	24	17.0	826	14.2
50–64 years	33	23.4	1390	23.8
65–84 years	42	29.8	2589	44.4
85+ years	28	19.9	529	9.1
Stage				
Localized	92	65.2	2912	49.9
Regional	23	16.3	542	9.3
Distant	5	3.5	112	1.9
Unknown	21	14.9	2266	38.9
Mode of diagnosis				
Cases microscopically verified	109	77.3	4322	74.1
Histology	109	77.3	4300	73.7
Cytology	0	0.0	22	0.4
Clinical examination or diagnosis (including radiography)	23	16.0	921	15.8
Unknown or other modes of diagnosis	9	6.4	589	10.1
Source of information				
Notification	139	98.6	5308	91.0
Death certificate only	2	1.4	524	9.0

The seven selected prefectures were Yamagata, Miyagi, Ibaraki, Fukui, Shiga, Osaka, and Fukuoka and together include approximately 18% of the Japanese population in 2014. Population denominators for rates were obtained from the National Cancer Center, Japan, which uses data published by the Statistics Bureau of Japan.^9^

### 2.2 Inclusion criteria

We included all cases of malignant brain and CNS tumors occurring among persons aged 20 and older in each data source based on the International Classification of Diseases for Oncology, 3rd edition (ICD‐O‐3) codes, according to groupings defined by the Central Brain Tumor Registry of the United States (Table S1).^3^ Tumors were classified as glioma, other specified malignant neoplasm, or unspecified malignant neoplasm. Within gliomas, tumors were subclassified as glioblastoma, all other astrocytic tumors, or other gliomas (Table S2).^3^ These subgroups were aggregated to balance granularity with reportability given the small number of cases among Japanese Americans. Lymphomas and hematopoietic neoplasms occurring in the CNS were excluded.

### 2.3 Statistical analysis

We estimated ASR per 100,000 person‐years using the direct method and the 2000 U.S. standard population.^11^ ASRs were estimated for the Japanese population living in the United States (Japanese American) and Japanese in Japan by CNS histologic subtype, sex, and age groups. For comparison, we also estimated ASR for NHW and AAPI in the United States. Incidence rates based on <10 cases per year were omitted. Since AAPI comprises an ethnically diverse group with distinct cultures, languages, diets, and cancer burden,^7^ we also compared ASRs between Japanese Americans and other Asian American/Pacific Islander subgroups available in SEER with at least 100 cases over the full time period: Chinese, Korean, Filipino, Vietnamese, and Asian Indian/Pakistani.

ASRs and 95% confidence intervals (95% CI) for the United States were calculated using SEER*Stat software (Version 8.6.1) and Stata software (version 16.0) for Japan.

## 3 RESULTS

The analyses presented are based on 26,210 malignant brain tumor cases among adults in the U.S. SEER‐9 registries and 5832 cases in Japan (Table 1). Of the U.S. cases, 1805 were among AAPI, including 141 cases (7.8%) reported among Japanese Americans. More than 50% of cases were male except for Japanese Americans (49.6%). The proportion of cases that were 65 and older was highest in Japan (53.2%) and among Japanese Americans (49.7%). Compared with Japanese Americans, NHW and AAPI, cases in Japan were more likely to be of unknown stage (38.9% vs. 8%–14.9% in the United States), to have an unknown method of diagnosis (10.1% vs. 3.4%–6.4% in the United States), or to be registered based on death certificate only (9.0% vs. <2%).

The ASR for malignant brain and CNS tumors among Japanese Americans (ASR: 2.56, 95% CI: 2.13–3.05) was lower than that observed in Japan (ASR: 3.66, 95% CI: 3.56–3.76) (Table 2). Both of these rates are substantially lower than the rate among the NHW population in the United States (ASR: 9.43 per 100,000, 95% CI: 9.31–9.56), as well as the overall AAPI population (ASR: 4.13, 95% CI: 3.94–4.33).

**TABLE 2:** Age‐standardized incidence rate (ASR)^a^ of malignant brain and CNS tumors by histology, 2007–2014.

Histology	Non‐Hispanic White^b^			Asian American/Pacific Islanders^b^		
	*N*	%	ASR (95% CI)	*N*	%	ASR (95% CI)
All malignant brain/CNS tumors	24,405	100	9.43 (9.31–9.56)	1805	100	4.13 (3.94–4.33)
All glioma	22,123	84.4	8.57 (8.46–8.69)	1541	85.4	3.51 (3.33–3.69)
Glioblastoma	14,510	55.4	5.29 (5.20–5.38)	882	48.9	2.04 (1.90–2.18)
All other astrocytic tumors	3673	14.0	1.57 (1.52–1.63)	290	16.1	0.66 (0.58–0.74)
Glioma, others	3940	15.0	1.71 (1.65–1.77)	369	20.4	0.81 (0.73–0.90)
Other specified malignant neoplasm	1067	4.1	0.44 (0.42–0.47)	166	9.2	0.37 (0.31–0.43)
Unspecified malignant neoplasm	1215	4.6	0.42 (0.39–0.44)	98	5.4	0.25 (0.21–0.31)

Histology	Japanese Americans^b^			Japan overall^c^		
	*N*	%	ASR (95% CI)	*N*	%	ASR (95% CI)
All malignant brain/CNS tumors	141	100	2.56 (2.13–3.05)	5832	100	3.66 (3.56–3.76)
All glioma	118	83.7	2.14 (1.75–2.59)	4159	71.3	2.67 (2.58–2.76)
Glioblastoma	65	46.1	1.07 (0.82–1.39)	2238	38.4	1.31 (1.26–1.37)
All other astrocytic tumors	28	19.9	0.54 (0.35–0.80)	783	13.4	0.56 (0.52–0.60)
Glioma, others	25	17.7	0.52 (0.33–0.79)	1138	19.5	0.80 (0.75–0.85)
Other specified malignant neoplasm	14	9.9	0.31 (0.16–0.52)	512	8.8	0.36 (0.32–0.39)
Unspecified malignant neoplasm	^d^	^d^	^d^	1161	19.9	0.63 (0.59–0.67)

^a^ Rates are per 100,000 and are age‐standardized to the 2000 United States standard population.

^b^ Data from SEER‐9 Registries.

^c^ Data from seven prefecture‐level populations registered in Japan.

^d^ Data withheld due to case counts <10.

**FIGURE 1:**
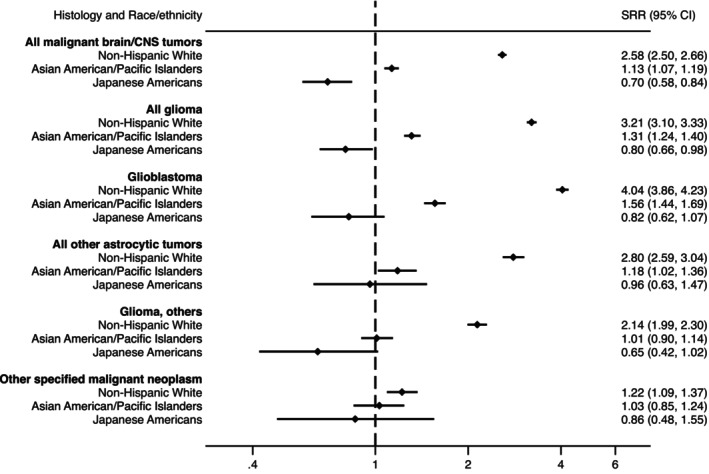
Standardized rate ratios for malignant brain and CNS tumors by race/ethnicity and histology relative to the Japan incidence rate, 2007–2014. *Histology and race/ethnicity groups with fewer than 10 cases were excluded.

The majority of cases in both Japanese and Japanese American populations were gliomas, with the lowest rates observed among Japanese Americans. Compared with Japan, the highest glioma rates were found among NHWs (Standardized Rate Ratio [SRR] = 3.21, 95% CI: 3.10–3.33) and the lowest among Japanese Americans (SRR = 0.80, 95% CI: 0.66–0.98) (Figure 1). This pattern persisted for glioblastomas and other gliomas, though the differences between racial and ethnic groups were less pronounced. Rates of other astrocytic tumors were relatively consistent across all groups except NHWs, who exhibited higher rates.

The pattern by sex mirrored the overall trend, with similar sex ratios observed across various racial and ethnic groups (Table 3). For all malignant brain and CNS tumors, gliomas, and glioblastomas, males exhibited 47%–67% higher incidence rates than females across the four comparison groups. Among Japanese Americans, there was a higher male‐to‐female incidence rate ratio for other astrocytic tumors and a lower ratio for other gliomas. However, these differences may be attributable to chance due to the small number of cases and wide confidence intervals.

**TABLE 3:** Age‐standardized incidence rate of malignant brain and CNS tumors by histology and sex, 2007–2014.

Histology	Male		Female		
	*N*	ASR (95% CI)	*N*	ASR (95% CI)	SRR (95% CI)^a^
All malignant brain/CNS tumors					
Non‐Hispanic White	13,907	11.41 (11.21, 11.60)	10,498	7.67 (7.52, 7.83)	1.49 (1.45, 1.53)
Asian American/Pacific Islanders	983	5.02 (4.70, 5.35)	822	3.42 (3.19, 3.66)	1.47 (1.34, 1.62)
Japanese Americans	70	3.22 (2.49, 4.10)	71	2.10 (1.60, 2.72)	1.54 (1.06, 2.22)
Japan overall	3173	4.35 (4.20, 4.51)	2659	3.05 (2.93, 3.18)	1.43 (1.35, 1.51)
All glioma					
Non‐Hispanic White	12,781	10.45 (10.26, 10.64)	9342	6.90 (6.76, 7.05)	1.51 (1.47, 1.56)
Asian American/Pacific Islanders	852	4.32 (4.03, 4.63)	689	2.86 (2.65, 3.08)	1.51 (1.36, 1.68)
Japanese Americans	59	2.71 (2.04, 3.53)	59	1.74 (1.28, 2.30)	1.56 (1.04, 2.35)
Japan overall	2380	3.24 (3.10, 3.38)	1779	2.17 (2.06, 2.29)	1.49 (1.39, 1.59)
Glioblastoma					
Non‐Hispanic White	8474	6.63 (6.49, 6.78)	6036	4.11 (4.01, 4.22)	1.61 (1.56, 1.67)
Asian American/Pacific Islanders	500	2.61 (2.38, 2.85)	382	1.59 (1.43, 1.76)	1.64 (1.43, 1.89)
Japanese Americans	31	1.40 (0.94, 2.01)	34	0.84 (0.56, 1.21)	1.67 (0.95, 2.89)
Japan overall	1304	1.65 (1.56, 1.74)	934	1.03 (0.96, 1.10)	1.60 (1.47, 1.76)
All other astrocytic tumors					
Non‐Hispanic White	2079	1.83 (1.75, 1.91)	1594	1.34 (1.28, 1.41)	1.36 (1.27, 1.46)
Asian American/Pacific Islanders	154	0.77 (0.65, 0.91)	136	0.57 (0.48, 0.67)	1.36 (1.07, 1.73)
Japanese Americans	16	0.77 (0.43, 1.27)	12	0.38 (0.18, 0.69)	2.06 (0.86, 5.09)
Japan overall	443	0.66 (0.60, 0.73)	340	0.47 (0.42, 0.53)	1.39 (1.20, 1.63)
Glioma, others					
Non‐Hispanic White	2228	1.99 (1.90, 2.07)	1712	1.44 (1.37, 1.52)	1.38 (1.29, 1.47)
Asian American/Pacific Islanders	198	0.94 (0.81, 1.08)	171	0.70 (0.60, 0.82)	1.34 (1.09, 1.66)
Japanese Americans	12	0.54 (0.27, 0.97)	13	0.52 (0.27, 0.92)	1.03 (0.41, 2.61)
Japan overall	633	0.93 (0.86, 1.01)	505	0.67 (0.61, 0.74)	1.39 (1.22, 1.57)
Other specified malignant neoplasm					
Non‐Hispanic White	591	0.51 (0.47, 0.56)	476	0.37 (0.34, 0.41)	1.38 (1.22, 1.57)
Asian American/Pacific Islanders	87	0.42 (0.33, 0.52)	79	0.32 (0.26, 0.40)	1.29 (0.93, 1.78)
Japanese Americans	^b^	^b^	^b^	^b^	^b^
Japan overall	262	0.40 (0.35, 0.46)	250	0.32 (0.28, 0.36)	1.27 (1.05, 1.53)
Unspecified malignant neoplasm					
Non‐Hispanic White	535	0.45 (0.41, 0.48)	680	0.40 (0.37, 0.43)	1.11 (0.99, 1.25)
Asian American/Pacific Islanders	44	0.28 (0.20, 0.38)	54	0.24 (0.18, 0.31)	1.20 (0.78, 1.82)
Japanese Americans	^b^	^b^	^b^	^b^	^b^
Japan overall	531	0.71 (0.65, 0.78)	630	0.56 (0.52, 0.61)	1.27 (1.12, 1.43)

^a^ SRR is defined as the incidence rate ratio between males and females.

^b^ Data withheld due to case counts <10.

The higher incidence rates in Japan compared with Japanese Americans persisted across all age groups for malignant brain tumors overall and gliomas (Table S3; Figures S1a,b). ASRs among NHW and AAPI in the United States were consistently higher than those in Japan for all age groups.

Within Asian American/Pacific Islanders, rates were lowest among Japanese Americans and highest among Asian Indian/Pakistani Americans, though all were lower than those of NHW (Table S4).

## 4 DISCUSSION

We conducted a comparative analysis of adult incidence rates of malignant brain and CNS tumors between Japan and various populations in the United States, including Japanese Americans, NHW, and AAPI. Our findings reveal that both Japanese and Japanese American populations exhibit the lowest incidence rates of brain and CNS tumors across most subtypes examined. This pattern remains consistent across different sex and age groups.

Recent international comparisons of brain tumor incidence, particularly between the United States and Japan, are sparse, posing challenges in contextualizing our findings within the existing literature. Nevertheless, our results indicate lower rates of brain tumors among Japanese American adults and residents of Japan. In contrast, reports from Asian countries indicate a higher incidence of CNS tumors among children, with the highest rates observed among children of Asian descent in the United States.^12^, ^13^, ^14^, ^15^

Certain inherited genetic variants that differ by ancestry can influence susceptibility to CNS tumors, such as gliomas and meningiomas. Differences in genes involved in DNA repair, cell cycle regulation, and immune response are associated with varying risks across populations.^16^, ^17^, ^18^, ^19^ Additionally, variations in tumor‐suppressor genes (like TP53) and oncogenes (like EGFR) may either increase susceptibility or provide protective effects against CNS tumors, contributing to incidence differences among racial and ethnic groups.^20^, ^21^ Further research is needed to elucidate the genetic factors driving variations in CNS tumors incidence among Japanese and other Asian populations, as well as between Asian and non‐Asian groups.

The observed pattern of risk is likely attributable to a complex interplay of various factors. A combination of health care type, genetic, viral, and data quality factors, among others, may explain the observed patterns in rates. For example, Japanese people could potentially have genetically reduced risk of brain and CNS tumors, but the increased use of diagnostic imaging in Japan could have resulted in improvements in diagnostic capabilities and consequently higher rates among Japanese in Japan than among Japanese Americans. In 2014, computed tomography (CT) and magnetic resonance imaging (MRI) scans per 1 million people were twice as high in Japan as in the United States.^22^ Unlike the United States, Japan has brain health checkups, which include CT and MRI diagnostic testing.^23^, ^24^ Given that our study is restricted to malignant tumors, the impact of these checkups on incidental diagnoses may be mitigated, but the scale of potential detection bias is unknown.

Viral factors may also play a role. Viruses have been linked to brain tumor risk, although the evidence is limited and has primarily concerned glioma.^14^ Specifically, Varicella zoster virus (VZV) has been consistently found to have a protective association with glioma whereas human cytomegalovirus (HCMV) has been suggested to be associated with increased glioma risk.^14^ Differing prevalence of these viruses in the United States and Japan could explain the lower risks among Japanese Americans than among Japanese in Japan. With respect to HCMV, there is wide variation in estimates of seroprevalence in the United States and Japan, making inference about the extent to which this might drive differential risks difficult.^25^ In terms of VZV, the United States introduced a vaccine in 1995, whereas Japan did not mandate VZV vaccination until 2014.^26^ While this nearly 20 year difference is large, it is unlikely that it would substantially impact our observations since vaccination usually happens in childhood and most of the cases of brain tumors we observed occurred in older age. Also, Japanese individuals who choose to emigrate may represent a healthier subset of the population, a phenomenon known as the “healthy immigrant effect.” As a result, these individuals may have fewer risk factors for brain cancer and consequently show a lower overall incidence of the disease. Additionally, although lifestyle factors such as dietary habits and healthcare practices may persist among Japanese Americans and potentially impact health outcomes, the influence of lifestyle factors on adult brain cancer incidence remains uncertain.^27^, ^28^, ^29^ Further exploration of these factors could provide valuable insights into cancer outcomes among individuals with Japanese ancestry.

Variations in cancer registration practices and data quality between Japan and the United States likely contribute to the observed differences in cancer rates. Similarly, within the United States, rates among Japanese Americans may be overestimated or underestimated due to numerator‐denominator bias. Numerator‐denominator bias would arise if the likelihood that a person is categorized as Japanese in the U.S. cancer registry differs from the likelihood that they will report Japanese ancestry in the U.S. census, which ultimately provides the numerator for the rates. Therefore, lower reporting of Japanese ethnicity in health records relative to the census in the United States could result in lower observed incidence rates in Japanese Americans compared with Japan overall. Other more weakly or inconsistently associated factors could also contribute to differences in brain tumor rates between these countries and ancestry groups. These include respiratory allergies, female sex hormones, and susceptibility genes in telomerase‐related pathways.^30^

Our study has several strengths. To the best of our knowledge, this is the first population‐based study utilizing nationally representative U.S. cancer registry data to investigate primary brain and CNS tumor rates among various AAPI subgroups, thereby addressing the limited understanding of brain tumor etiology and risk variation in this diverse racial and ethnic group. Second, by building on the same principles as migration studies, our comparison may help generate hypotheses as to the relative impact and interplay of genetic and environmental risk factors in these populations. Third, utilizing comprehensive registry data from Japan enables us to better understand cancer incidence by subtype and sex within the Japanese population and to make more accurate comparisons with the incidence rates in the United States.

However, study limitations should be acknowledged. First, the rates in Japan were not restricted to people of Japanese ancestry, so the rates represent the overall population rather than in a single ancestry group. However, in 2014, only 1.6% of the population was foreigners in these seven prefectures, so this is unlikely to have had a major impact.^31^ Second, we do not have information about whether Japanese American individuals immigrated or were descendants of immigrants, so we cannot infer how risk changes upon migration. However, our study aligns with other international comparisons that evaluate individuals of the same ancestry across different countries.^32^, ^33^ Third, while we focused on high‐quality registries, the higher proportion of unspecified histology cases in Japan could limit histology‐specific comparisons. Despite these challenges, there is nevertheless value in comparing the incidence of brain tumors internationally and by ethnicity since the etiology of these tumors remains largely unexplained.

In migrant studies, if incidence rates among migrants tend to approach those of the host country, environmental factors may be more likely to play a role. However, we did not observe this pattern: Japanese Americans had much lower rates compared with NHW in the United States, who comprise most of the host country population. Incidence rates among Japanese Americans mirror the lower rates observed in Japan. This finding is consistent with a strong role of genetic factors in brain tumor etiology, highlighting the need for further research examining key drivers. International, ethnically diverse genetic studies may be necessary to identify key genetic pathways.

Results from this study revealed that the incidence rates of adult brain and CNS tumors among Japanese Americans followed the lower rates in Japan, which were lower than those observed in the host country. Improving cancer registration in Japan and improving race/ethnicity‐specific data collection in the United States will be crucial in further elucidating these patterns and understanding the interplay between genetic and environmental factors.

## AUTHOR CONTRIBUTIONS

**Byron Sigel:** Conceptualization; investigation; writing – original draft; methodology; writing – review and editing; visualization; validation; formal analysis. **Diana R. Withrow:** Validation; investigation; writing – original draft; conceptualization; methodology; writing – review and editing. **Lene H. S. Veiga:** Validation; writing – review and editing; supervision; methodology; conceptualization; investigation. **Eiko Saito:** Supervision; writing – review and editing; conceptualization; investigation; methodology. **Tomohiro Matsuda:** Funding acquisition; project administration; resources; data curation; writing – review and editing. **Kota Katanoda:** Supervision; funding acquisition; validation; project administration; resources; data curation; software; writing – review and editing; methodology; conceptualization.

## FUNDING INFORMATION

This study was supported by a Health Labour Sciences Research Grant of the Ministry of Health, Labour and Welfare of Japan (20EA1026, 20EA1017, and 23EA1009).

## CONFLICT OF INTEREST STATEMENT

The authors have no conflict of interest to disclose.

## ETHICS STATEMENT

The study using the MCIJ data was approved by the Internal Review Board of the National Cancer Center, Japan (2019‐202).

## Supporting information


**Data S1.** Supporting Information.

## Data Availability

The data supporting the results of this study are available upon reasonable request to the corresponding author, with permission from the ACCCC Committee for Medical and Health Research.
