# Supplementary material for: Adult brain cancer incidence patterns: A comparative study between Japan and Japanese Americans
Source: Int J Cancer. 2025 Feb 21;157(3):436–44. doi: 10.1002/ijc.35374 (PMC12141977; doi:10.1002/ijc.35374)
Supplement: Supplementary file 1 — Data S1. Supporting Information. [file IJC-157-436-s001.pdf]

Supplementary

Adult brain cancer incidence patterns: A comparative study between Japan and Japanese Americans

Byron Sigel, Diana R. Withrow, Lene H.S.Veiga, Eiko Saito, Tomohiro Matsuda,

Kota Katanoda

Contents:

Supplementary Tables

i. Supplementary Table 1
ii. Supplementary Table 2
iii. Supplementary Table 3
iv. Supplementary Table 4

Supplementary Figures

i. Supplementary Figure 1a
ii. Supplementary Figure 1b

Supplementary Tables

Supplementary Table 1. Classification of brain and other CNS tumors by ICD-O-3 topography codes*

**Site**	**ICD-O-3[b] Topography**
Cerebrum	C71.1
Frontal lobe of brain	C71.2
Temporal lobe of brain	C71.3
Parietal lobe of brain	C71.4
Occipital lobe of brain	C71.5
Ventricle	C71.6
Cerebellum	C71.7
Brain stem	C71.8-C71.9
Other brain	C71.8
Overlapping lesion of brain	C71.9
Brain, NOS	C72.0-C72.1
Spinal cord and cauda equina	C72.0
Spinal cord	C72.1
Cauda equine	C72.2-C72.5
Cranial nerves	C72.2
Olfactory nerve	C72.3
Optic nerve	C72.4
Acoustic nerve	C72.5
Cranial nerve, NOS	C72.8-C72.9
Other nervous system	C72.8
Overlapping lesion of brain and central nervous system	C72.9
Nervous system, NOS	C70.0-C70.9
Meninges (cerebral & spinal)	C70.0
Cerebral meninges	C70.1
Spinal meninges	C70.9
Meninges, NOS	C75.1-C75.2
Pituitary and craniopharyngeal duct	C75.1
Pituitary gland	C75.2
Craniopharyngeal duct	C75.3
Pineal gland	C30.0†

Classification obtained from CBTRUS Statistical Report: Primary Brain and Other Central Nervous System Tumors Diagnosed in the United States in 2012–2016

*International Classification of Diseases for Oncology, 3rd Edition, 2000. World Health Organization, Geneva, Switzerland

†ICD-O-3 histology codes 9522–9523 only

Supplementary Table 2. Classification of malignant brain and CNS by ICD-O-3 morphology codes based on WHO Classification of Central Nervous System Tumors

Site Group	ICD-O-3[a] Topography
All Glioma	9380-9384, 9391-9460
Glioblastoma	9440, 9441, 9442
All other astrocytic tumors	9381, 9384, 9400, 9401, 9410, 9411, 9420, 9421, 9424
Glioma, others	9380, 9382, 9383, 9391, 9392, 9393, 9394, 9430, 9444, 9450, 9541, 9460
Other specified malignant neoplasm	8011, 8020, 8041, 8046, 8050, 8052, 8070, 8140, 8200, 8246, 8260, 8270, 8272, 8280, 8300, 8322, 8400, 8440, 8481, 8500, 8680, 8693, 8710, 8711, 8720, 8728, 8761, 8770, 8800, 8801, 8802, 8803, 8804, 8805, 8806, 8810, 8815, 8830, 8840, 8850, 8890, 8894, 8900, 8910, 8920, 8963, 9040, 9041, 9044, 9060, 9061, 9064, 9065, 9070, 9071, 9080, 9084, 9085, 9100, 9101, 9120, 9130, 9133, 9150, 9161, 9180, 9220, 9231, 9240, 9242, 9260, 9282, 9350, 9362, 9364, 9370, 9371, 9372, 9390, 9470, 9471, 9472, 9473, 9474, 9490, 9500, 9501, 9503, 9505, 9506, 9508, 9522, 9523, 9530, 9531, 9532, 9533, 9537, 9538, 9539, 9540, 9560, 9561, 9580
Unspecified malignant neoplasm	8000-8005, 8010, 8021

Supplementary Table 3. Age-standardized incidence rate of malignant brain and other CNS tumors by histology and age, 2007-2014

	20-39 years			40-64 years		
**Histology**	N	%*	ASR (95% CI)	N	%	ASR (95% CI)
All malignant brain/CNS tumors						
Non-Hispanic White	2,952	100	4.14 (3.99, 4.29)	10,712	100	9.05 (8.87, 9.23)
Asian American/Pacific Islander	353	100	1.88 (1.69, 2.08)	768	100	3.65 (3.40, 3.92)
Japanese Americans	16	100	1.19 (0.68, 1.93)	55	100	2.59 (1.93, 3.39)
Japan Overall	783	100	1.99 (1.85, 2.13)	1,922	100	3.27 (3.12, 3.43)
All Glioma						
Non-Hispanic White	2,636	89.3	3.71 (3.57, 3.85)	10,089	94.2	8.51 (8.34, 8.69)
Asian American/Pacific Islander	297	84.1	1.58 (1.41, 1.77)	690	89.8	3.28 (3.04, 3.54)
Japanese Americans	11	68.8	0.82 (0.41, 1.46)	49	89.1	2.31 (1.70, 3.08)
Japan Overall	589	75.2	1.48 (1.37, 1.61)	1,536	79.9	2.62 (2.48, 2.76)

	65+ years		
**Histology**	N	%	ASR (95% CI)
All malignant brain/CNS tumors			
Non-Hispanic White	10,741	100	22.21 (21.79, 22.64)
Asian American/Pacific Islander	684	100	10.33 (9.56, 11.13)
Japanese Americans	70	100	5.56 (4.28, 7.11)
Japan Overall	3,127	100	8.33 (8.04, 8.63)
All Glioma			
Non-Hispanic White	9,408	87.6	19.61 (19.21, 20.01)
Asian American/Pacific Islander	554	81.0	8.35 (7.67, 9.08)
Japanese Americans	58	82.9	4.67 (3.50, 6.12)
Japan Overall	2,034	65.0	5.45 (5.21, 5.69)

Rates are per 100,000 and are age-standardized to the 2000 United States standard population
*Percentages represent the proportion of a specific type of tumor in relation to all malignant brain and central nervous system (CNS) tumors within each defined race/ethnicity and age group.

Supplementary Table 4. Age-standardized incidence rate of malignant brain and CNS tumors by histology and Asian American/Pacific Islander Groups, 2007-2014

	**Chinese**			**Korean**		
**Histology**	N	%	ASR (95% CI)	N	%	ASR (95% CI)
All malignant brain/CNS tumors	420	100	3.85 (3.49, 4.25)	129	100	3.20 (2.65, 3.82)
All Glioma	351	83.6	3.21 (2.88, 3.57)	108	83.7	2.60 (2.12, 3.15)
Glioblastoma	200	47.6	1.83 (1.58, 2.10)	61	47.3	1.52 (1.15, 1.96)
All other astrocytic tumors	61	14.5	0.56 (0.43, 0.72)	16	12.4	0.39 (0.22, 0.63)
Glioma, others	90	21.4	0.82 (0.66, 1.01)	31	24.0	0.70 (0.47, 1.00)
Other specified malignant neoplasm	39	9.3	0.36 (0.25, 0.49)	11	8.5	0.26 (0.13, 0.47)
Unspecified malignant neoplasm	30	7.1	0.29 (0.20, 0.42)	10	7.8	0.34 (0.16, 0.61)

	**Filipino**			**Vietnamese**		
**Histology**	N	%	ASR (95% CI)	N	%	ASR (95% CI)
All malignant brain/CNS tumors	399	100	3.87 (3.49, 4.28)	191	100	4.60 (3.95, 5.34)
All Glioma	330	82.7	3.13 (2.80, 3.50)	164	85.9	3.94 (3.33, 4.62)
Glioblastoma	197	49.4	1.85 (1.59, 2.13)	92	48.2	2.29 (1.82, 2.83)
All other astrocytic tumors	63	15.8	0.62 (0.48, 0.80)	36	18.8	0.85 (0.58, 1.19)
Glioma, others	70	17.5	0.66 (0.51, 0.84)	36	18.8	0.80 (0.56, 1.13)
Other specified malignant neoplasm	44	11.0	0.43 (0.31, 0.58)	19	9.9	0.45 (0.26, 0.71)
Unspecified malignant neoplasm	25	6.3	0.30 (0.20, 0.45)	8	4.2	0.22 (0.08, 0.44)

	**Asian Indian/Pakistani**			**Japanese Americans**		
**Histology**	N	%	ASR (95% CI)	N	%	ASR (95% CI)
All malignant brain/CNS tumors	359	100	6.80 (6.04, 7.64)	141	100	2.56 (2.13, 3.05)
All Glioma	333	92.8	6.38 (5.64, 7.2)	118	83.7	2.14 (1.75, 2.59)
Glioblastoma	196	54.6	4.30 (3.67, 5.01)	65	46.1	1.07 (0.82, 1.39)
All other astrocytic tumors	55	15.3	0.86 (0.62, 1.16)	28	19.9	0.54 (0.35, 0.80)
Glioma, others	82	22.8	1.22 (0.95, 1.56)	25	17.7	0.52 (0.33, 0.79)
Other specified malignant neoplasm	20	5.6	0.29 (0.17, 0.47)	14	9.9	0.31 (0.16, 0.52)
Unspecified malignant neoplasm	6	1.7	0.13 (0.04, 0.30)	†	†	†

Rates are per 100,000 and are age-standardized to the 2000 United States standard population
Other Asian American/Pacific Islander groups were not included due to the small number of cases (<100 cases)
†Data withheld due to case counts <10

Supplementary Figures

Supplementary Figure 1a. Standardized rate ratios for all malignant brain and CNS tumors by race/ethnicity and age, 2007–2014

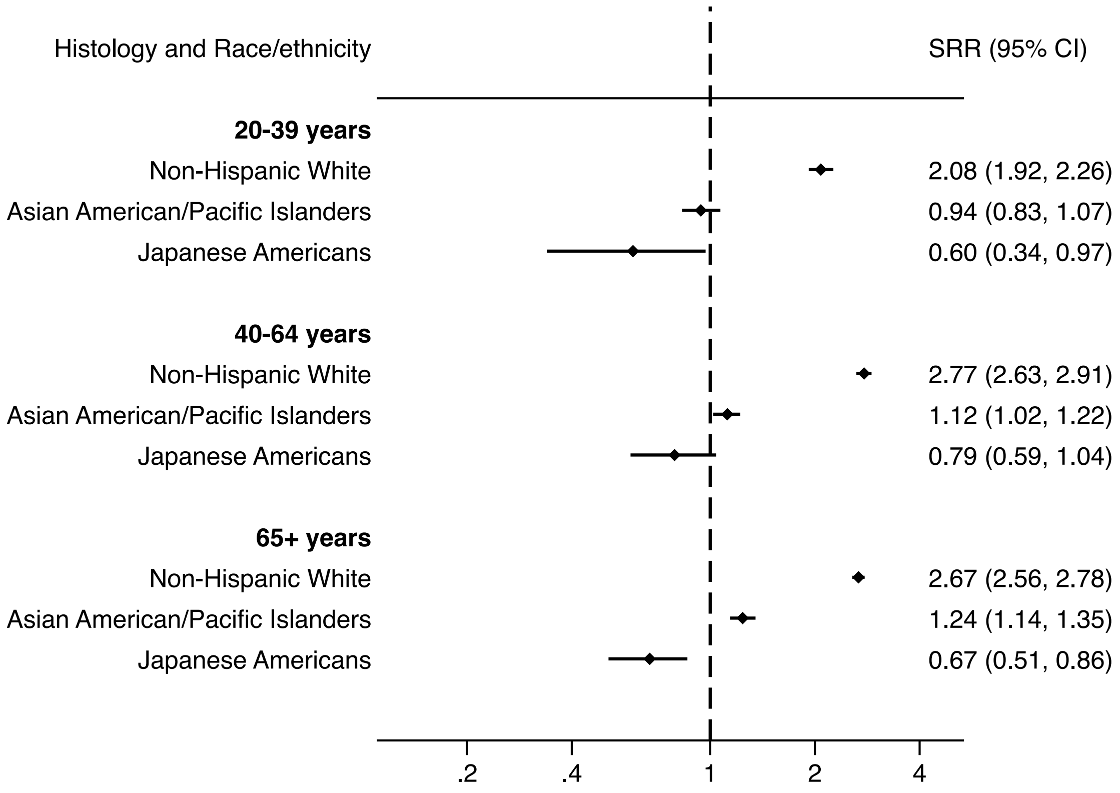

Standardized Rate Ratios (SRRs) relative to Japan's incidence rate (2007–2014)

Supplementary Figure 1b. Standardized rate ratios for all glioma by race/ethnicity and age, 2007–2014

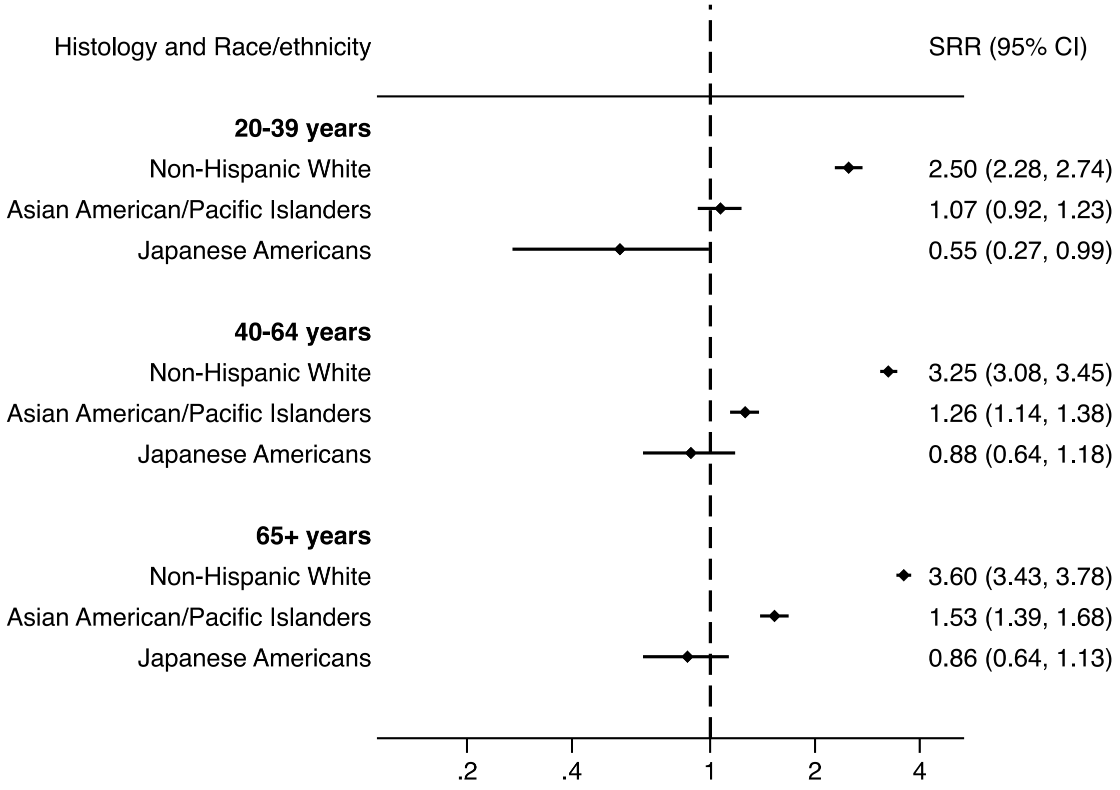

Standardized Rate Ratios (SRRs) relative to Japan's incidence rate (2007–2014)
